# Physical activity interests and preferences of cancer patients with brain metastases: a cross-sectional survey

**DOI:** 10.1186/s12904-016-0083-x

**Published:** 2016-01-19

**Authors:** Sonya S. Lowe, Brita Danielson, Crystal Beaumont, Sharon M. Watanabe, Kerry S. Courneya

**Affiliations:** Department of Symptom Control and Palliative Care, Cross Cancer Institute, 11560 University Avenue, Edmonton, Alberta T6G 1Z2 Canada; Division of Radiation Oncology, Department of Oncology, University of Alberta, Cross Cancer Institute, 11560 University Avenue, Edmonton, Alberta T6G 1Z2 Canada; Division of Palliative Care Medicine, Department of Oncology, University of Alberta, Cross Cancer Institute, 11560 University Avenue, Edmonton, Alberta T6G 1Z2 Canada; Faculty of Physical Education and Recreation, University of Alberta, 1-113 University Hall, Edmonton, Alberta T6G 2H9 Canada

**Keywords:** Cancer, Brain metastases, Physical activity, Preferences, Survey, Palliative care

## Abstract

**Background:**

Physical activity has been shown to positively impact cancer-related fatigue, physical functioning and quality of life outcomes in early stage cancer patients, however its role at the end stage of cancer has yet to be determined. Brain metastases are amongst the most common neurological complications of advanced cancer, with significant deterioration in fatigue and quality of life. The purpose of the present study was to examine the physical activity interests and preferences of cancer patients with brain metastases initiating palliative whole brain radiotherapy.

**Methods:**

Thirty-one patients aged 18 years or older, cognitively intact, diagnosed with brain metastases, and with Palliative Performance Scale scores of greater than 30 %, were recruited from a multidisciplinary outpatient brain metastases clinic. An interviewer-administered survey was used to assess physical activity interests and preferences of participants who were embarking upon palliative whole brain radiotherapy.

**Results:**

87 % (*n* = 27) of participants felt that physical activity was important, however there was limited interest in participating in a structured program at the onset of palliative whole brain radiotherapy. Lung cancer diagnosis was associated with being less interested in participating in a physical activity program, and feeling less able to participate in a physical activity program at the onset of palliative whole brain radiotherapy.

**Conclusions:**

Cancer patients with brain metastases demonstrate limited interest and varied preferences for physical activity during palliative whole brain radiotherapy. Additional pilot work with this patient population is needed before physical activity interventions can be tested in clinical research.

## Background

Increased disease and symptom burden contribute to poor quality of life for patients with metastatic cancer [1]. Brain metastases are the most frequent neurologic sequelae of cancer, with up to 40 % of all cancer patients developing brain metastases over the course of their illness; lung cancer is the most common primary site [2]. Whole brain palliative radiotherapy is the primary treatment for cancer patients with multiple brain metastases, with positive effects on survival and local disease control [3]. In a prospective study of 151 patients with previously untreated brain metastases, there was significant deterioration in fatigue, physical functioning, motor dysfunction, leg weakness and global quality of life at 3 months post-whole brain palliative radiotherapy [4].

A recent Cochrane review has shown that physical activity interventions can improve fatigue, physical functioning and quality of life outcomes in early stage cancer patients both during and following active treatment [5]. The role of physical activity in addressing quality of life outcomes in cancer patients with brain metastases, however, is unknown. Prior to designing any physical activity intervention in this patient population, knowledge of their specific physical activity interests and preferences may enhance recruitment, adherence and optimize potential benefits and desired outcomes [6].

Numerous studies have examined the physical activity preferences of cancer patients [7–10], but only one has focused on patients with advanced cancer [11], and none have examined cancer patients with brain metastases. The physical activity preferences of cancer patients with early disease stage and good prognosis may be very different than cancer patients with brain metastases because of substantial disease burden, functional limitations, symptom burden, and psychosocial distress in the latter group [12].

We previously reported a study examining the associations between objectively-measured physical activity, quality of life, physical functioning and symptoms in cancer patients with brain metastases who were undergoing palliative whole brain radiotherapy; complete details regarding the parent study setting, participants and procedures have been reported elsewhere [13]. The present article reports the physical activity interests and preferences of this sample of cancer patients with brain metastases undergoing palliative whole brain radiotherapy. Our hypotheses were that a majority of this sample of brain metastases patients would be interested and able to participate in a physical activity intervention, and similar to the advanced cancer population [11], the majority would be interested in walking and home-based interventions.

## Methods

### Setting and participants

Ethical approval was obtained from the Health Research Ethics Board of Alberta Health Services to recruit potential participants from the multidisciplinary Rapid Access Palliative Radiotherapy Program (RAPRP) brain metastases clinic [14] at the Cross Cancer Institute in Edmonton, Canada, between November 2009 and June 2011. Eligible participants read and signed a consent form, which detailed the risks and benefits of participating, confidentiality, and the right to withdraw from the study. Of 111 patients who were approached regarding the study, 31 (28 %) patients consented to and completed the survey interview; the most common reason for exclusion or declining to participate was cognitive impairment. All participants were diagnosed with advanced cancer, had radiological confirmation of brain metastases, and were undergoing palliative whole brain radiotherapy. Consecutive patient referrals were screened for eligibility criteria including: 1) 18 years of age or older; 2) ability to understand, provide written informed consent in, and speak English; and 3) cognitive ability to participate (defined as a normal Folstein’s Mini Mental State Score for patient’s age and education level [15]). Participants were deemed ineligible if they presented with: 1) any absolute contraindications to physical activity [16]; or 2) Palliative Performance Scale (PPS) level of 30 % or less [17]. The PPS tool measures performance status in palliative care across five domains including ambulation, activity and evidence of disease, self-care, intake and level of consciousness; the PPS has been widely used and validated in advanced cancer populations [18]. 

The study was a cross-sectional, interviewer-administered questionnaire. After the survey, each participant wore an activPAL™ accelerometer (PAL Technologies Ltd., Glasgow, UK) to record triaxial movement [19] for up to 7 days of whole brain palliative radiotherapy treatment. For the purposes of this study, physical activity was defined as any bodily movement produced by the skeletal muscles that results in a substantial increase in energy expenditure over resting levels [20]. Physical activity program preferences were elicited by seven open short items and four closed short items, all of which were drawn from previous research in advanced cancer patients [11]. Participants were asked to complete items regarding program preferences even if they were not interested in a physical activity program. Participants were asked about the importance of physical activity to them, their perceived ability to participate in a physical activity program, their favorite physical activity and what type of physical activity they were most interested in. Participants were asked to select their preferences regarding physical activity from the following four categories: company (alone, with caregiver/spouse, with family/friends, with other cancer patients, no preference), location (at home, at a hospital-based center, at a cancer center, at a local fitness center, no preference), time of day (morning, afternoon, evening, no preference), and duration (less than 10 min, 10-20 min, 20-30 min, over 30 min, not at all). Participants were asked to respond to the following seven items in open-question format: the meaning of physical activity to them, the current importance of being physically active, current interest in a physical activity program, self-assessment of current ability to participate in a physical activity program, frequency of desired physical activity program, favorite physical activity, and type of physical activity most interested in currently. Descriptive statistics were used to analyze and categorize open-item responses under main themes, which were identified in common by the participants.

### Statistical analysis

Data were analyzed using SPSS version 22.0 software (SPSS, Inc., Evanston, IL). Frequency counts and percentages were calculated to determine the physical activity interests and preferences of the sample. Chi-square analysis was performed to explore potential associations between physical activity preferences and the following medical, demographic and physical activity variables: age (<60 years versus ≥60 years), gender (male versus female), body mass index (normal/underweight versus overweight/obese), Palliative Performance Scale level (<60 % versus ≥60 %), total number of comorbidities (<2 versus ≥2), survival from time of interview to time of death (<90 days versus ≥90 days), and current time spent sitting or supine per day (<20.7 h versus ≥20.7 h). Cut-points were based on a roughly median split of the data, as described previously [13]. All statistical tests were two-sided (α = 0.05).

## Results

Details regarding sample demographic and medical characteristics have been reported elsewhere [13]. Given that this was a pilot study, a total of 31 participants were recruited. Participants’ mean age was 63.5 ± 10.4 years, 58 % were female (*n* = 18), 74 % were married or in a common-law relationship (*n* = 23), 58 % completed grade 12 education or higher (*n* = 18), and the majority were not employed (*n* = 24, 77 %). The average body mass index was 27.5 (SD 4.9, *n* = 31). Lung cancer was the most common diagnosis (*n* = 19, 61 %). The complete Edmonton Symptom Assessment System (ESAS) scores for this sample were reported elsewhere [13], but in brief: the ESAS pain score was 1.3 ± 1.8, fatigue score was 4.2 ± 2.7, anxiety score was 3.5 ± 2.9, and feeling of well-being score was 2.6 ± 2.0. 62 % (*n* = 19) of participants had a Palliative Performance Scale level of 80 % or higher, and median survival was 171 days from time of study consent.

### Physical activity preferences

Details of the participants’ physical activity preferences are presented in Table 1. 65 % (*n* = 20) of participants reported that they would not be interested in a physical activity program at the onset of palliative whole brain radiotherapy, with 58 % (*n* = 18) of participants reporting that they did not feel able to participate in a physical activity program. When asked about preferences if they were to do a physical activity program, 29 % (*n* = 9) of participants preferred to participate in a physical activity program with family or friends, with 19 % (*n* = 6) of participants indicating a preference to do so with their spouse or caregiver. Moreover, 58 % (*n* = 18) of participants indicated that they would prefer to do a physical activity program in their own homes and 52 % (*n* = 16) reported a preference to do a physical activity program in the morning. Preferring to perform a physical activity program of between 20 to 30 min in duration, and between two to three times per week, was endorsed by 36 % (*n* = 11) of participants. Finally, 48 % (*n* = 15) of participants indicated that walking was the type of physical activity in which they were most interested at the time of survey.

**Table 1:** Descriptive statistics for physical activity preferences of study participants (n = 31)

Preference variable	N (%)
Is being physically active important to you now?	
Yes	27(87 %)
No	4(13 %)
Are you interested in a physical activity program now?	
Yes	6(19 %)
No	20(65 %)
Maybe	5(16 %)
Do you think you would be able to participate in a physical activity program now?	
Yes	12(39 %)
No	18(58 %)
Maybe	1(3 %)
If you were to begin a physical activity program, who would you like to participate with?	
Alone	5(16 %)
With caregiver/spouse	6(19 %)
With family/friends	9(29 %)
With other cancer patients	2(7 %)
No preference	9(29 %)
If you were to begin a physical activity program, where would you like to participate?	
At home	18(58 %)
At a hospital-based center	0
At a cancer center	0
At a local fitness center	5(16 %)
No preference	8(26 %)
If you were to begin a physical activity program, would you prefer to participate in the:	
Morning	16(52 %)
Afternoon	8 (26 %)
Evening	2(7 %)
No preference	5(16 %)
If you were to begin a physical activity program, how long do you think you would be able to participate?	
< 10 minutes	7(23 %)
10 to 20 minutes	9(29 %)
20 to 30 minutes	11(36 %)
> 30 minutes	3(10 %)
If you were to begin a physical activity program, how often would you be interested in participating?	
Once per week	1(3 %)
2 to 3 times per week	11(36 %)
Once per day	9(29 %)
Other	10(32 %)
What is your favorite physical activity?	
Walking	15(48 %)
Golfing	3(10 %)
Swimming	2(6 %)
Cycling/Stationary Bike Riding	2(6 %)
Driving	2(6 %)
Other	7(23 %)
What type of physical activity would you be most interested in now?	
Walking	14(45 %)
Golfing	2(6 %)
Treadmill	2(6 %)
Swimming	2(6 %)
Other	10(32 %)
None	1(3 %)

In response to the open-ended question regarding their perceived meaning of physical activity, 45 % (*n* = 14) of participants reported “being mobile”, 14 % (*n* = 4) reported “exercise”, 10 % (*n* = 3) reported “being healthy”, and 10 % (*n* = 3) reported “housework”. The majority of participants who reported no interest in a physical activity program, indicated that “symptom management issues” were the primary deterrent.

### Associations between demographic, medical and physical activity variables and physical activity preferences

Details of the associations between demographic, medical and physical activity variables and physical activity preferences are presented in Tables 2 and 3. Chi-square analyses indicated that having a lung cancer diagnosis was associated with being less interested in participating in a physical activity program (Fisher’s exact, *p* < 0.01) and feeling less able to participate in a physical activity program (Fisher’s exact, *p* < 0.01) at the onset of palliative whole brain radiotherapy. The remaining medical and demographic variables (i.e., age, body mass index, total number of comorbidities, Palliative Performance Scale level, and survival from time of survey to time of death) and median time spent sitting or supine over the past week did not influence physical activity preferences in this sample.

**Table 2:** Associations between demographic variables and physical activity preferences in cancer patients with brain metastases (n = 31)

Group	Preference	Association
Male versus Female	Less interested in physical activity program now	(8 % vs. 28 %; Fisher’s exact, *p* = 0.36)
	More able to participate in physical activity program now	(46 % vs. 33 %; *χ* ^2^ = 0.12, *p* = 0.73)
	Less likely to participate with caregiver/spouse, friends/family	(39 % vs. 56 %;*χ* ^2^ = 0.33, *p* = 0.57)
	More likely to participate at home	(69 % vs. 50 %; *χ* ^2^ = 0.49, *p* = 0.48)
	Less likely to participate in the morning	(46 % vs. 56 %; *χ* ^2^ = 0.02, *p* = 0.88)
	Less likely to participate in for 20 minutes or more	(58 % vs. 39 %; *χ* ^2^ = 0.45, *p* = 0.50)
	More likely to participate 2 to 3 times per week or more	(69 % vs. 61 %; Fisher’s exact, *p* = 0.72)
	Less likely to prefer walking as favorite physical activity	(31 % vs. 61 %; *χ* ^2^ = 1.70, *p* = 0.19)
	Less likely to prefer walking as physical activity most interested in now	(39 % vs. 64 %; *χ* ^2^ = 0.07, *p* = 0.79)
60 years or older versus	More interested in physical activity program now	(20 % vs. 18 %; Fisher’s exact, *p* = 1.00)
Younger than 60	More able to participate in physical activity program now	(40 % vs. 36 %; Fisher’s exact, *p* = 1.00)
	Less likely to participate with caregiver/spouse, friends/family	(35 % vs. 73 %; *χ* ^2^ = 2.68, *p* = 0.10)
	More likely to participate at home	(70 % vs. 36 %; Fisher’s exact, *p* = 0.13)
	Less likely to participate in the morning	(45 % vs. 64 %; *χ* ^2^ = 0.38, *p* = 0.54)
	Less likely to participate in for 20 minutes or more	(42 % vs. 55 %; *χ* ^2^ = 0.08, *p* = 0.78)
	More likely to participate 2 to 3 times per week or more	(65 % vs. 64 %; Fisher’s exact, *p* = 1.00)
	More likely to prefer walking as favorite physical activity	(60 % vs. 27 %; *χ* ^2^ = 1.87, *p* = 0.17)
	More likely to prefer walking as physical activity most interested in now	(55 % vs. 27 %; Fisher’s exact, *p* = 0.26)
Normal/Underweight versus	Less interested in physical activity program now	(0 % vs. 27 %); Fisher’s exact, *p* = 0.15)
Overweight/Obese	Less able to participate in physical activity program now	(11 % vs. 50 %; Fisher’s exact, *p* = 0.10)
	More likely to participate with caregiver/spouse, friends/family	(67 % vs. 41 %; Fisher’s exact, *p* = 0.25)
	More likely to participate at home	(67 % vs. 55 %; Fisher’s exact, *p* = 0.70)
	Less likely to participate in the morning	(44 % vs. 55 %; Fisher’s exact, *p* = 0.70)
	Less likely to participate in for 20 minutes or more	(33 % vs. 52 %; Fisher’s exact, *p* = 0.44)
	More likely to participate 2 to 3 times per week or more	(67 % vs. 64 %; Fisher’s exact, *p* = 1.00)
	Less likely to prefer walking as favorite physical activity	(44 % vs. 50 %; Fisher’s exact, *p* = 1.00)
	Less likely to prefer walking as physical activity most interested in now	(33 % vs. 50 %; Fisher’s exact, *p* = 0.46)
Less than 20.7 hours versus	More interested in physical activity program now	(42 % vs. 8 %; Fisher’s exact, *p* = 0.16)
20.7 hours or more spent	Less able to participate in physical activity program now	(42 % vs. 58 %; *χ* ^2^ = 0.17, *p* = 0.68)
sitting or supine per day	Less likely to participate with caregiver/spouse, friends/family	(50 % vs. 50 %; *χ* ^2^ = 0.00, *p* = 1.00)
	Less likely to participate at home	(50 % vs. 58 %; *χ* ^2^ = 0.00, *p* = 1.00)
	More likely to participate in the morning	(75 % vs. 42 %; *χ* ^2^ = 1.54, *p* = 0.21)
	Less likely to participate in for 20 minutes or more	(42 % vs. 67 %; *χ* ^2^ = 0.67, *p* = 0.41)
	Less likely to participate 2 to 3 times per week or more	(58 % vs. 83 %; Fisher’s exact, *p* = 0.37)
	More likely to prefer walking as favorite physical activity	(67 % vs. 42 %; *χ* ^2^ = 0.67, *p* = 0.41)
	More likely to prefer walking as physical activity most interested in now	(50 % vs. 42 %; *χ* ^2^ = 0.00, *p* = 1.00)

**Table 3:** Associations between medical variables and physical activity preferences in cancer patients with brain metastases (n = 31)

Group	Preference	Association
Lung cancer versus	Less interested in physical activity program now	(0 % vs. 50 %; Fisher’s exact, *p* = 0.001)
Other cancer	Less able to participate in physical activity program now	(16 % vs. 75 %; Fisher’s exact, *p* = 0.002)
	Less likely to participate with caregiver/spouse, friends/family	(47 % vs. 50 %; *χ* ^2^ = 0.00, *p* = 1.00)
	More likely to participate at home	(84 % vs. 17 %; *χ* ^2^ = 11.15, *p* = 0.001)
	More likely to participate in the morning	(53 % vs. 50 %; *χ* ^2^ = 0.00, *p* = 1.00)
	Less likely to participate in for 20 minutes or more	(39 % vs. 58 %; *χ* ^2^ = 0.45, *p* = 0.50)
	Less likely to participate 2 to 3 times per week or more	(63 % vs. 67 %; Fisher’s exact, *p* = 1.00)
	Less likely to prefer walking as favorite physical activity	(48 % vs. 58 %; *χ* ^2^ = 0.26, *p* = 0.60)
	Less likely to prefer walking as physical activity most interested in now	(42 % vs. 50 %; *χ* ^2^ = 0.00, *p* = 0.95)
2 or more versus	Less interested in physical activity program now	(6 % vs. 36 %; Fisher’s exact, *p* = 0.07)
Less than 2 comorbidities	Less able to participate in physical activity program now	(35 % vs. 43 %; *χ* ^2^ = 0.00, *p* = 0.95)
	More likely to participate with caregiver/spouse, friends/family	(59 % vs. 36 %; *χ* ^2^ = 0.85, *p* = 0.36)
	Less likely to participate at home	(53 % vs. 64 %; *χ* ^2^ = 0.07, *p* = 0.79)
	More likely to participate in the morning	(53 % vs. 50 %; *χ* ^2^ = 0.00, *p* = 1.00)
	Less likely to participate in for 20 minutes or more	(41 % vs. 54 %; *χ* ^2^ = 0.10, *p* = 0.75)
	More likely to participate 2 to 3 times per week or more	(71 % vs. 57 %; Fisher’s exact, *p* = 0.48)
	Less likely to prefer walking as favorite physical activity	(41 % vs. 57 %;*χ* ^2^ = 0.28, *p* = 0.60)
	More likely to prefer walking as physical activity most interested in now	(47 % vs. 43 %; *χ* ^2^ = 0.00, *p* = 1.00)
Less than 60 % versus	Less interested in physical activity program now	(0 % vs. 20 %; Fisher’s exact, *p* = 1.00)
60 % or greater	Less able to participate in physical activity program now	(0 % vs. 40 %; Fisher’s exact, *p* = 1.00)
Palliative Performance Scale	More likely to participate with caregiver/spouse, friends/family	(100 % vs. 47 %; Fisher’s exact, *p* = 0.48)
level	More likely to participate at home	(100 % vs. 57 %; Fisher’s exact, *p* = 1.00)
	More likely to participate in the morning	(100 % vs. 50 %; Fisher’s exact, *p* = 1.00)
	Less likely to participate in for 20 minutes or more	(0 % vs. 48 %; Fisher’s exact, *p* = 1.00)
	More likely to participate 2 to 3 times per week or more	(100 % vs. 63 %; Fisher’s exact, *p* = 1.00)
	Less likely to prefer walking as favorite physical activity	(0 % vs. 50 %; Fisher’s exact, *p* = 1.00)
	Less likely to prefer walking as physical activity most interested in now	(0 % vs. 47 %; Fisher’s exact, *p* = 1.00)
Less than 90 days versus	Less interested in physical activity program now	(7 % vs. 31 %; Fisher’s exact, *p* = 0.15)
90 days or greater survival	Less able to participate in physical activity program now	(33 % vs. 39 %; Fisher’s exact, *p* = 1.00)
from time of interview	More likely to participate with caregiver/spouse, friends/family	(47 % vs. 46 %; *χ* ^2^ = 0.00, *p* = 1.00)
to time of death	More likely to participate at home	(67 % vs. 54 %; *χ* ^2^ = 0.09, *p* = 0.76)
	Less likely to participate in the morning	(47 % vs. 62 %; *χ* ^2^ = 0.17, *p* = 0.68)
	More likely to participate in for 20 minutes or more	(43 % vs. 39 %; *χ* ^2^ = 0.00, *p* = 1.00)
	More likely to participate 2 to 3 times per week or more	(67 % vs. 54 %; *χ* ^2^ = 0.09, *p* = 0.76)
	Less likely to prefer walking as favorite physical activity	(40 % vs. 54 %; *χ* ^2^ = 0.12, *p* = 0.72)
	More likely to prefer walking as physical activity most interested in now	(47 % vs. 39 %; *χ* ^2^ = 0.00, *p* = 0.96)

## Discussion

Although the small sample size precludes drawing conclusions regarding associations, this study highlights the limited and varied physical activity interests and preferences of cancer patients with brain metastases who are undergoing palliative whole brain radiotherapy. 87 % of participants in this sample indicated that physical activity was important to them. However, the majority of participants in this sample reported that they would not be interested in (65 %) and did not feel able to participate (58 %) in a physical activity program at the time of palliative whole brain radiotherapy.

These contradictory findings are unique amongst previous research of physical activity preferences in other groups of cancer patients. For example, in a population-based survey of 600 colorectal cancer survivors, 78 % of respondents were interested in, and 84 % of respondents felt able to participate in, a physical activity program [10]. In a population-based survey of over 700 kidney cancer survivors, over 80 % of respondents indicated that they would be interested and felt able to do a physical activity program [9]. Finally, in a population-based survey of 359 ovarian cancer survivors, 54 % of respondents indicated interest in, and 65 % felt capable of, participating in a physical activity [8]. Poorer prognosis, greater disease burden and higher symptom profiles in cancer patients with brain metastases may explain the differences in perceived interest in physical activity, and perceived ability to participate in a physical activity program, compared to other cancer populations.

In an institution-based survey of 106 primary brain cancer patients, 45 % of respondents were interested in, and 47 % of respondents reported that they felt able to, participate in a physical activity program during adjuvant treatment, which included surgical resection and chemotherapy; in contrast, 84 % of respondents felt able to participate in physical activity after completion of adjuvant therapy [7]. Compared to primary brain malignancy, the higher disease burden and symptom profile of patients with metastatic spread of cancer to the brain, may explain these differing preferences.

With regards to their favorite physical activity, 48 % of participants in this current sample endorsed walking, with 45 % of participants indicating that walking was the type of physical activity in which they were most interested. Given the disease burden and symptom profile of advanced cancer patients, walking may be the most feasible option because it does not require any equipment, does not incur significant cost, and is not predicated on previous physical activity history. If they were to participate in a physical activity program, 49 % of this current brain metastases sample preferred doing physical activity with a spouse, caregiver, family or friends. Given that 62 % (*n* = 19) of participants had a Palliative Performance Scale level of 80 % or higher, this shift away from engaging in physical activity alone may suggest a need for emotional, rather than physical, support from those who are closest to them.

With respect to preferred site of programming, 58 % of the current sample of cancer patients with brain metastases would prefer to engage in a physical activity program in their own homes; there was no reported interest in participating in a structured program at a hospital-based or cancer center. Given that these participants were embarking on daily palliative whole brain radiotherapy treatments at the time of survey, this finding may suggest a desire to engage in physical activity outside the medical treatment setting.

Chi-square analyses yielded a negative association between diagnosis of lung cancer, and interest in participating in a physical activity program and feeling able to participate in a physical activity program at the onset of palliative whole brain radiotherapy. Nineteen (61 %) of the thirty-one patients in our sample had a lung cancer diagnosis, which is reflective of the fact that up to 40 % of non-small cell lung carcinoma patients develop brain metastases over the course of their illness [21]. The small sample size precludes drawing inferences or making recommendations about cancer specific physical activity programs; larger studies are required to confirm these results and further delineate potential interactions between medical, demographic, behavioral variables and physical activity preferences in cancer patients with brain metastases.

Study limitations include the fact that this current sample may not be representative of all cancer patients with brain metastases, given the functional status and prognosis required to embark on and tolerate palliative whole brain radiotherapy on an outpatient basis. The cross-sectional nature of this study precludes examination of whether the physical activity preferences of brain metastases may change during the course of palliative whole brain radiotherapy of after its completion. Our survey did not specifically elicit patient barriers to physical activity which may have aided in our understanding of why a structured program was not the preferred modality. As this was a pilot study, further statistical analysis is limited by our small sample size. Moreover, our small sample size precludes establishing relationships between physical activity preferences and specific symptoms, as well as demographic and medical variables.

## Conclusions

The results of this study highlight the limited interest and varied physical activity preferences of cancer patients with brain metastases initiating palliative whole brain radiotherapy. Although there is strong endorsement of the importance of physical activity, the majority of participants in this sample did not feel willing or able to participate in a physical activity program at the onset of palliative whole brain radiotherapy. It is unclear if cancer patients with brain metastases are uninterested in any physical activity program at any time, or whether they are just uninterested in a structured physical activity program during palliative whole brain radiotherapy. Longitudinal studies are needed to determine whether these interests and programming preferences change both during and after palliative whole brain radiotherapy. Future studies are needed to compare the physical activity interests and preferences of cancer patients with brain metastases who do not undergo palliative whole brain radiotherapy, and ultimately, to determine whether physical activity interventions are even feasible in this patient population.
